# Survival of Patients With Metastatic Rectum Cancer Who Underwent Metastasectomy Following Conversion Chemotherapy Sans Pelvic Radiotherapy: A Turkish Oncology Group Study

**DOI:** 10.7759/cureus.39119

**Published:** 2023-05-17

**Authors:** Elvina Almuradova, Suayib Yalcin, Rukiye Arıkan, Murat Ayhan, Hacer Demir, Gokcen Tugba Cevik, Mustafa Karaca, Ibrahim Petekkaya, Bulent Karabulut

**Affiliations:** 1 Medical Oncology, Ege University Hospital, Izmir, TUR; 2 Medical Oncology, Hacettepe University Medical School, Ankara, TUR; 3 Medical Oncology, Marmara University School of Medicine, Istanbul, TUR; 4 Medical Oncology, Dr. Lutfi Kırdar Kartal Education and Research Hospital, Istanbul, TUR; 5 Medical Oncology, Afyonkarahisar University of Health and Science, Afyonkarahisar, TUR; 6 Medical Oncology, Afyon Kocatepe University School of Medicine, Afyonkarahisar, TUR; 7 Medical Oncology, Usak Education and Research Hospital, Usak, TUR; 8 Medical Oncology, Antalya Education and Research Hospital, Antalya, TUR; 9 Medical Oncology, Tınaztepe University Hospital, Izmir, TUR

**Keywords:** radiotherapy, chemotherapy, metastasectomy, metastatic, rectum cancer

## Abstract

Background: The management of early rectal cancer is different from that of colon cancer in terms of radiotherapy (RT) requirements or neoadjuvant treatment. It is not clear how the course of rectal cancer differs from that of the colon in a metastatic setting or how it should be approached differently. This study aimed to evaluate outcomes after combining downsizing chemotherapy (CTx) with rescue surgery.

Methods: Eighty-nine patients (57 men and 32 women) diagnosed with metastatic rectal cancer with resectable disease after systemic CTx were included in the study. All patients underwent surgery for the primary mass and metastasis, but none received radiation therapy before or after surgery. Survival curves for overall survival (OS) and progression-free survival (PFS) were generated using the Kaplan-Meier method and compared with the log-rank test for subgroups.

Results: The median follow-up time was 28.8 (17.6-39.4) months. During the follow-up, 54 (60.7%) patients died and 78 (87.6%) patients had a PFS event. Cancer relapsed in 72 (80.9%) patients. Median OS was 35.2 (95% CI: 28.5-41.8) months, and median PFS was 17.7 (95% CI: 14.4-21) months. The five-year OS and PFS were 19% and 3.5%, respectively. Male sex (p=0.04) and a better Mandard score (p=0.021) were associated with a longer OS, while obesity was associated with a shorter PFS (p<0.001).

Conclusion: Our study is the first to evaluate the effects of metastasectomy after conversion therapy in metastatic rectal cancer independent of colon cancer. As a result of the study, it was seen that the survival after metastasectomy in rectal cancer is worse than the colon cancer data known from previous studies.

## Introduction

Colorectal cancer (CRC) is the second most common cancer in women and the third most common cancer in men [1]. Unfortunately, 21% to 33.7% of CRC patients are diagnosed with liver metastasis [2]. Even with the most potent chemotherapy (CTx) agents, five-year survival is only up to 4% to 20% in patients with metastatic CRC. However, survival may increase by up to 30% to 35% in patients who have had successful surgery after systemic anticancer therapy [3].

Although rectal cancer is generally considered CRC because it is the second most common tumor of the large intestine, there are distinct differences in its epidemiology, disease course, and treatment protocol [4]. There are established guidelines for treating early-stage rectal cancer and the role of pelvic radiotherapy (RT) [5,6]. However, there is no consensus on pelvic irradiation in patients with resectable metastatic disease after CTx. It is also unknown whether metastasectomy outcomes differ from those of colon cancer, as the data of rectal cancer patients who have undergone metastasectomy has not been separately evaluated. As the Turkish Oncology Study Group (TOG), we aimed to assess the survival of patients diagnosed with rectal cancer who underwent metastasectomy after conversion CTx but did not receive local RT.

## Materials and methods

Patient selection

This is a retrospective cohort study approved by the Institutional Review Board of Ege University Hospital (approval no. 20-2.1T/14). It included 89 patients diagnosed with metastatic rectal cancer from six different centers in Turkey who were followed up between 2012 and 2020. Inclusion criteria for the study were: 1) a diagnosis of metastatic rectal cancer, 2) no primary cancer of another organ, 3) having an unresectable primary disease at initial diagnosis, 4) having undergone surgery for metastases and primary diseases after a favorable response to CTx, and 5) not having received local RT. Patients with local RT, non-resectable metastatic disease after CTx, early-stage disease, and other primary malignancies were excluded.

Evaluation and treatment

After diagnosing metastatic rectal cancer and determining the tumor resectability by a multidisciplinary team, conversion CTx was initiated. All patients received two to 21 cycles of the upfront systemic CTx with folinic acid, fluorouracil (5-FU), and irinotecan (FOLFIRI); or 5-FU, leucovorin, and oxaliplatin (FOLFOX); or oxaliplatin and capecitabine (XELOX) based regimen with or without bevacizumab or cetuximab or panitumumab. Patients underwent surgery for the primary mass and metastases after systemic treatment. Maintenance CTx was commenced for 55 patients after surgery.

Follow-up and survival analysis

Patients were followed up at three- or six-month intervals postoperatively. They were evaluated with routine blood tests, carcinoembryonic antigen (CEA), and imaging studies at each visit and were examined for treatment-related complications. To analyze the cohort, demographic characteristics (age, gender, BMI) and comorbid diseases of the patients were recorded. Preoperative histopathological differentiation degree and a pathological component of the tumor, KRAS, NRAS, BRAF molecular analyzes, tumor localization, treatment regimen, surgery type, tumor regression degree (according to Mandard), and the place and time of relapse were obtained from the hospital files [7]. The primary outcome of the study was to evaluate overall survival (OS) and progression-free survival (PFS) in patients with advanced rectal cancer who did not receive local RT and underwent metastasectomy after successful conversion CTx. The secondary outcome was to reveal demographic, clinical, and pathological factors associated with OS and PFS in this group.

Statistical analysis

All analyses were performed with SPSS Statistics, version 25 (IBM Corp., Armonk, NY, USA). Descriptive statistics were presented with frequency (%) and median (IQR). The OS time was defined as the period from diagnosis to the last follow-up and/or death, and the PFS time was defined as the period between diagnosis to disease progression and/or death. Survival analyses were carried out using the Kaplan-Meier method, and a comparison of prognostic subgroups was performed with log-rank tests. A 5% type-I error level was used to infer statistical significance.

## Results

Baseline characteristics

A total of 89 patients (57 men and 32 women) with a median age of 59 (54-68) were recruited. Thirty (33.7%) patients were 65 years of age or older. The median BMI was 59 (54-68) kg/m2 and 14 (15.7%) patients had a BMI of ≥30 kg/m2. A total of 45 (50.6%) patients had comorbid disease(s), and the most common comorbidities were hypertension (40.4%) and diabetes mellitus (22.5%). Moderate differentiation was the most frequently observed histological grade (44.9%). Mucinous and signet ring cell histology were observed in 15 (16.9%) and nine (10.1%) patients, respectively. The KRAS was positive in 33 (46.5%) of 71 patients, and NRAS was positive in 16 (23.5%) of 68 patients. The BRAF and high microsatellite instability (MSI-H) were evaluated in only 38 (one positive) and 13 (all negative) patients, respectively. Primary cancer was located in the upper rectum/rectosigmoid region in 35 (39.3%), and in the lower rectum/sphincter region in 54 (60.7%) patients. At the time of diagnosis, 51 (57.3%) patients had liver metastasis, 12 (13.5%) patients had lung metastasis, and 30 (33.7%) patients had other organ metastasis. As conversion therapy, 20 (22.5%) patients received only systemic CTx, 47 (52.8%) patients received CTx plus bevacizumab, 14 (15.7%) patients received CTx plus cetuximab, and eight (9%) patients received CTx plus panitumumab. The most common surgical methods for the primary mass were low anterior resection (62.9%) and abdominoperineal resection (20.2%). On postoperative histopathological examination, tumor regression grade according to Mandard was 1, 2, or 3 in 20 (22.5%) patients, and 4 or 5 in nine (10.1%) patients. A total of 55 (61.8%) patients received maintenance therapy. Patient characteristics are presented in Table 1.

**Table 1:** Patient characteristics MSI-H: High microsatellite instability, CTx: Chemotherapy, TRG: Tumour regression grade

Characteristics	Frequency (%), n=89
Male sex	57 (64)
Age | Median (IQR) years (age ≥65 years)	59 (54-68) | 30 (33.7)
BMI | Median (IQR) kg/m^2 ^(BMI ≥30 kg/m^2^)	26.1 (24.3-29) | 14 (15.7)
Comorbid diseases: Hypertension | Diabetes mellitus | Coronary artery disease | Inflammatory bowel disease | Others	Total: 45 (50.6) | 36 (40.4) | 20 (22.5) | 8 (9.0) | 1 (1.1) | 9 (10.1)
Pathological differentiation degree: Well | Moderate | Poor | Unspecified	13 (14.6) | 40 (44.9) | 13 (14.6) | 23 (25.8)
Pathological component: Mucinous | Signet ring cell | Unspecified	15 (16.9) | 9 (10.1) | 65 (73)
KRAS positivity, n=71	33 (46.5)
NRAS positivity, n=68	16 (23.5)
BRAF positivity, n=38	1 (2.6)
MSI-H positivity, n=13	0 (0)
Localization: Upper/rectosigmoid | Lower/sphincter	35 (39.3) | 54 (60.7)
Metastasis site: Liver | Lung | Other	51 (57.3) | 12 (13.5) | 30 (33.7)
Conversion therapy: Systemic CTx | only CTx plus bevacizumab | CTx plus cetuximab | CTx plus panitumumab	20 (22.5) | 47 (52.8) | 14 (15.7) | 8 (9)
Type of surgery: Low anterior resection | Abdominoperineal resection | Proctocolectomy | Hartmann's procedure | Other | Unspecified	56 (62.9) | 18 (20.2) | 2 (2.2) | 2 (2.2) | 5 (5.6) | 6 (6.7)
Mandard score: TRG 1, 2, or 3 | TRG 4 or 5 | Unspecified	20 (22.5) | 9 (10.1) | 60 (67.4)
Maintenance therapy: Yes | No | Unspecified	55 (61.8) | 23 (25.8) | 11 (12.4)

Survival analysis

**Figure 1:**
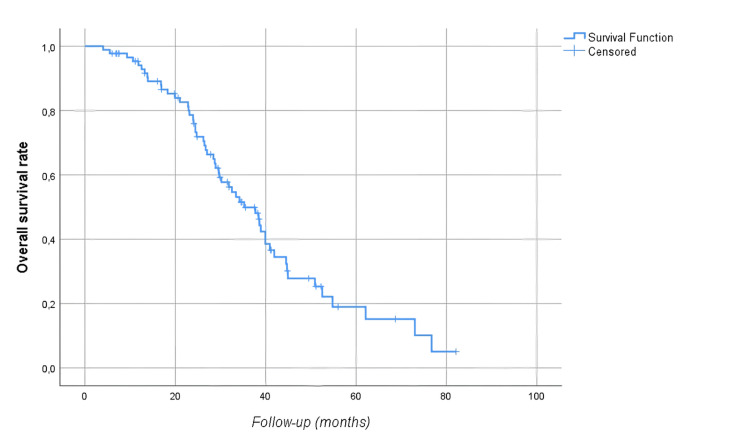
Overall survival rates of the study cohort

**Figure 2:**
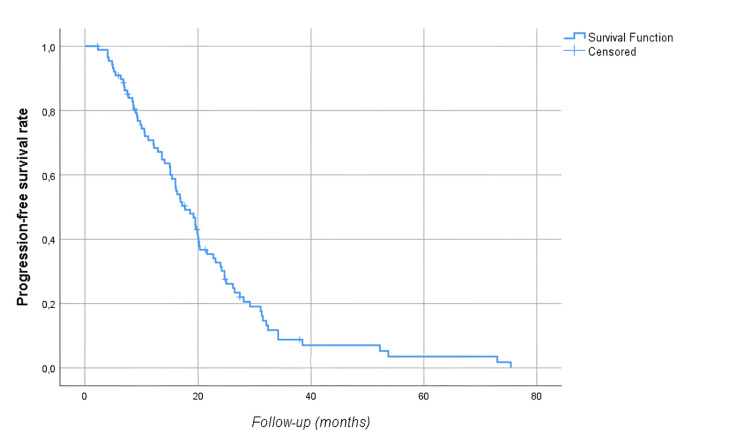
Progression-free survival rates of the study cohort

**Figure 3:**
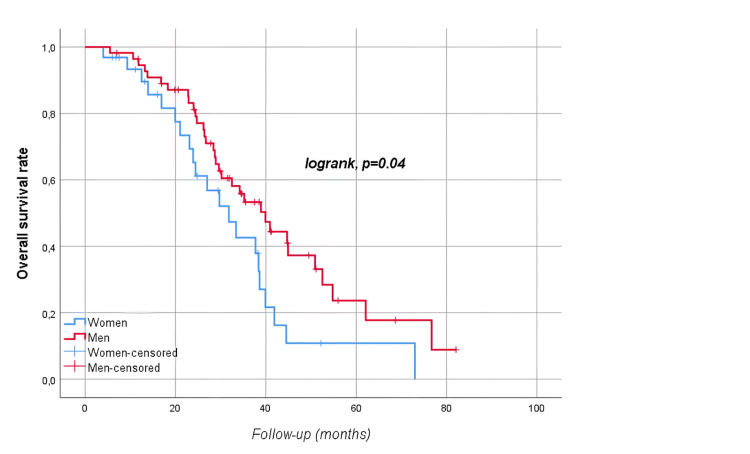
Overall survival rates by gender

**Figure 4:**
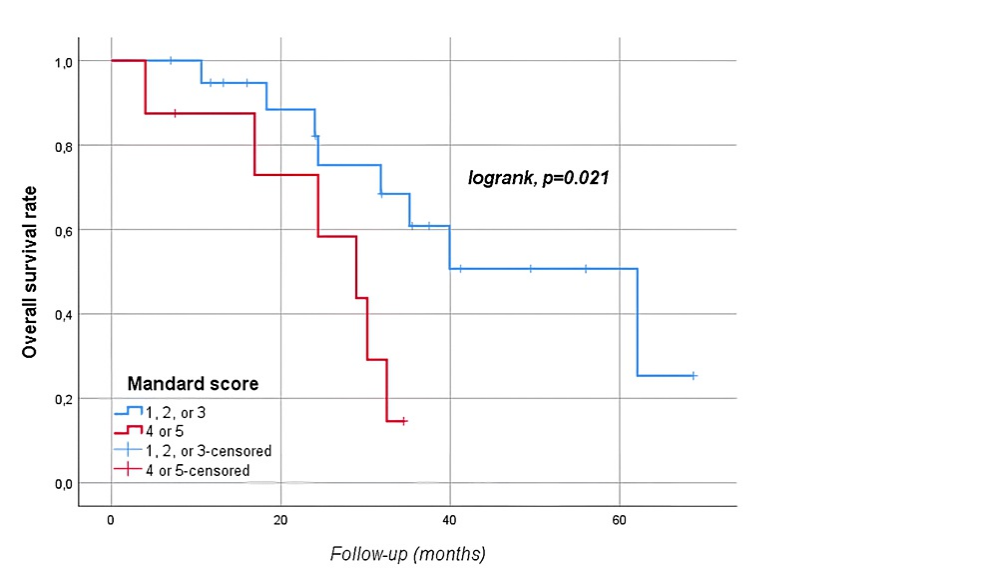
Overall survival rates by Mandard score

**Figure 5:**
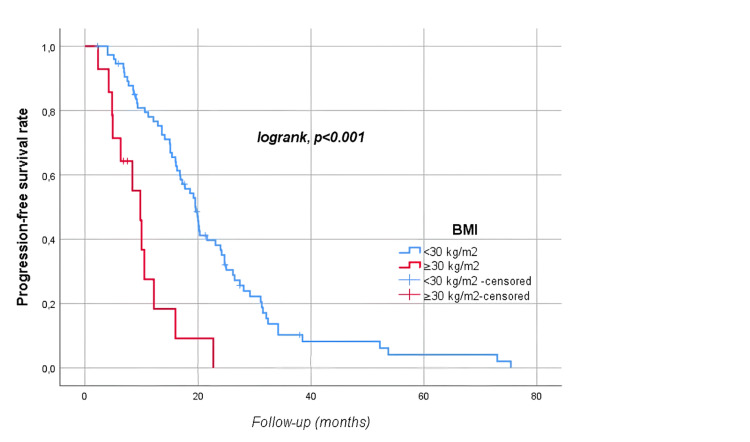
Progression-free survival rates by body mass index

The median follow-up time was 28.8 (17.6-39.4) months. At follow-up, 54 (60.7%) patients died, and 78 (87.6%) patients had disease progression. Median OS was 35.2 (95% CI: 28.5-41.8) months, and median PFS was 17.7 (95% CI: 14.4-21) months. The five-year OS and PFS of the patients were 19% and 3.5%, respectively (Figures 1-2). Median OS was significantly higher in men than in women (39.9 vs. 31.8 months, p=0.04) as shown in Figure 3. There was no statistically significant relationship between OS with age (≥ vs. <65 years, p=0.67), BMI (≥ vs. <30 kg/m2, p=0.226), presence of comorbidity (p=0.338), degree of pathological differentiation (p=0.943), pathological component (p=0.515), KRAS (p=0.117) or NRAS (p=0.881) positivity, primary tumor localization (p=0.568), metastasis site (liver only vs. others, p=0.845), type of systemic anticancer therapy (CTx plus anti-vascular endothelial growth factor (VEGF) vs. CTx plus anti-epidermal growth factor receptor (EGFR), p=0.478), and administration of maintenance therapy (p=0.668). According to Mandard, better tumor regression grade (1, 2, or 3 vs. 4 or 5) was associated with longer OS (62.1 vs. 28.9 months, p=0.021) as displayed in Figure 4. The PFS was compatible with OS, except for gender (p=0.431), BMI (p<0.001), and Mandard score (p=0.933) (Table 2). It was revealed that patients with a BMI ≥30 kg/m2 had a significantly lower PFS than those with a BMI <30 kg/m2 (9.8 vs. 19.5 months, p<0.001 as shown in Figure 5.

**Table 2:** Median OS and PFS logrank tests OS: Overall survival, PFS: Progression-free survival, SRC: Signet ring cell, CTx: Chemotherapy, VEGF: Vascular endothelial growth factor, EGFR: Epidermal growth factor receptor, TRG: Tumor regression grade

Parameters	Groups	Median PFS		Median OS	
		Months	p-value	Months	p-value
Sex	Men | Women	19.2 | 16	0.431	39.9 | 31.8	0.04
Age	≥65 years | <65 years	17.7 | 17.2	0.854	33.4 | 37.7	0.670
BMI	≥30 kg/m^2^ | <30 kg/m^2^	9.8 | 19.5	<0.001	31.8 | 37.7	0.226
Comorbidity	Present | Absent	15.4 | 19.5	0.912	40.9 | 32.5	0.338
Pathological differentiation degree	Well | Moderate | Poor	19.2 | 19.6 | 10.6	0.474	31.8 | 38.6 | 24.4	0.943
Pathological component	Mucinous | SRC	16.8 | 20.3	0.574	29.6 | 35.2	0.515
KRAS	Positive Negative	16.8 | 16	0.196	27 | 37.7	0.117
NRAS	Positive | Negative	23.1 | 16	0.297	33.4 | 34.2	0.881
Localization	Upper/rectosigmoid | Lower/sphincter	20.3 | 16.3	0.211	38.9 | 32.5	0.568
Metastasis site	Liver only | Others	16.8 | 15.1	0.776	32.5 | 38.6	0.845
Conversion therapy	CTx + anti-VEGF | CTx + anti-EGFR	19.2 | 16.9	0.387	31.8 | 35.2	0.478
Mandard score	TRG 1, 2, or 3 | TRG 4 or 5	16.8 | 19.2	0.933	62.1 | 28.9	0.021
Maintenance therapy	Yes | No	14.1 | 24	0.118	32.5 | 34.2	0.668

Recurrence features

Cancer recurred in 72 (80.9%) patients during follow-up. Of these, two (2.8%) had only local recurrence, 62 (86.1%) had only distant recurrence, and eight (11.1%) had both local and distant recurrence. The most common sites of distant recurrence were the liver (30.6%) and lung (25%) (Table 3). Patients who developed only liver relapse had similar OS (p=0.351) and PFS (p=0.573) rates as all other relapsed patients.

**Table 3:** Recurrence sites

Localization	Frequency (%), n=72
Local recurrence	2 (2.8)
Distant recurrence	62 (86.1)
Liver	22 (30.6)
Lung	18 (25)
Liver and lung	11 (15.3)
Other	6 (8.3)
Liver and other	5 (6.9)
Local and distant recurrence	8 (11.1)

## Discussion

Surgery could be considered for patients with limited metastatic disease burden [8,9]. Patients who were able to undergo resection for isolated metastases have improved survival or even a chance for a cure [10]. Although it has different biology and different approaches in the early stage, metastatic rectal cancer is evaluated under the title of CRC. Therefore, it is not known whether survival differs from colon cancer in patients with rectal cancer who underwent metastasectomy after conversion therapy. Our study is the first multicenter study evaluating survival in patients diagnosed with rectal cancer who underwent metastasectomy.

In studies evaluating colon cancer patients who underwent metastasectomy after response to CTx the median OS was about 40% to 60%. In a similar study conducted with a small patient group in our country, the five-year survival rate was detected to be as high as 49% in patients with CRC who underwent pulmonary metastasectomy [11]. Giacchetti et al. reported a 50% (range: 38-61) five-year OS rate in patients who were initially unresectable but had surgery with curative intent following neoadjuvant 5-FU/FA/oxaliplatin-based treatment [12]. The median survival of patients was 48 months in their study (range: 25-71) [12]. In the study by Fernandez et al., five-year survival after resection of hepatic metastases from CRC in selected patients screened by positron emission tomography with F-18 fluorodeoxyglucose was as high as 60% [13]. In these studies, the five-year PFS is seen to be between 22% and 30%. In our study, the median OS was 35.2 (95% CI: 28.5-41.8) months, the five-year OS rate was 19%, and the five-year PFS was only 3.5% (Figures 1-2).

Our study demonstrated that the survival rate for primary rectal carcinoma was lower than known historic data. However, when we look at some studies with subgroup analysis, we see that patients with primary cancer in the rectum have less survival in accordance with our study data. Rene et al. found that primary tumors originating from the rectum adversely affected survival, and five-year OS in these patients was 25% vs. 36% in patients with colon primary (p=0.08) [14]. In another study by French researchers, it has been shown that the five-year OS of rectal cancer patients who underwent metastasectomy was almost two times less than that of colon cancer patients (19% vs. 42%, p=0.006) [15]. All these study results support our data.

Previous studies did not find any difference in survival by gender [16,17]. However, this study found that men survived better (mOS 39.9 vs. 31.8 months, p=0.04, Figure 3). Chiang et al.'s research showed that patients with low-rectal cancer had significantly worse five-year OS and PFS rates (47.25% and 44.07%, respectively) than patients with mid-rectal cancer (63.46% and 60.22%, respectively) and upper-rectal cancer (73.91% and 71.87%, respectively) [18]. These findings were confirmed in the study by Huang et al., which showed that upper rectum cancer has similar PFS and a trend towards longer OS compared with those who had middle or lower rectal lesions [19]. Despite the fact that patients with lower rectum cancer had numerically inferior survival in our study, this difference was not statistically significant. In contrast to patients who received surgery for pulmonary-only CRC metastases, Landes et al. showed that patients who previously developed liver metastases have a higher risk of tumor recurrence and poorer survival [20]. In their study, Kim et al. compared the five-year OS and disease-free survival rates between CRC patients who underwent lung and hepatic metastasectomy and those who underwent lung metastasectomy alone [21]. They found no statistically significant difference between the two groups. In their sample of 29 patients, Mineo et al. were unable to demonstrate that the simultaneous occurrence of lung and hepatic metastases was a significantly poor prognostic factor [22]. Our study findings are in line with the latest studies, as there was no significant survival difference in the outcomes of hepatic and pulmonary metastasectomies.

The KRAS mutations in colon cancers have been associated with poorer survival and increased tumor aggressiveness [23]. In our study, no significant association was found between the RAS mutation and the survival of rectum cancer patients. The decision to choose a biological agent to be given in addition to CTx in colon cancer is made according to the tumor's side. It is known that the addition of anti-VEGF agents in the right colon and anti-EGFR agents in the left colon provides a better survival advantage in studies conducted so far [24]. Since rectal cancer is generally evaluated under the name of CRC, the information about which biological agent should be combined with CTx in this cancer is not clear. In our study, biological agents combined with CTx did not have a significant effect on survival.

It is known that the Mandard system provides higher accuracy for tumor regression in predicting rectal cancer prognosis when neoadjuvant conventional RT is applied [25]. The prognostic significance of the degree of pathological regression for metastatic lymph nodes after RT has also been demonstrated [26]. Although metastatic disease was evaluated in our study, the degree of primary tumor regression was found to have a positive effect on OS (p=0.021, Figure 4).

Research results regarding the relationship between BMI and survival in CRC are contradictory. Although some studies indicate that there is an increased risk of death from all causes with a high BMI, some studies on the contrary report that a high BMI is a good survival factor [27,28]. In our research, patients with a BMI ≥30 kg/m2 had a significantly lower PFS than p<0.001 (Figure 5).

As locoregional recurrence can be extremely morbid, prevention of recurrence by the addition of preoperative or postoperative CTx and/or RT remains a valuable target for early-stage rectal cancer. For metastatic disease, there is no clear information on this subject, and RT is not applied even if many patients respond very well to CTx and have been operated on, as in our study. The reason for this is that it is more important to choose the treatment modality that provides systemic control in metastatic disease [29]. As a matter of fact, studies conducted so far have shown that distant recurrence is more common in this group of patients and that pelvic RT does not have significant effects on survival [28-30]. In one of these studies, 185 patients with rectal cancer with synchronous resectable liver metastases were examined, and the systemic sites involved in the development of metastases were overwhelmingly more common than pelvic recurrences (<5% vs. 70%) [30]. In another study, although pelvic RT numerically reduced local recurrence, this was not statistically significant and did not contribute to overall survival [31]. In our study, in accordance with previous studies, it was observed that recurrent disease was mostly in the form of distant metastases and mostly in the liver. The presence of recurrent disease in the liver or lung did not make any difference in survival.

## Conclusions

For the first time, in our study, the effects of metastasectomy after conversion therapy in metastatic rectal cancer were evaluated independently of colon cancer. As a result of the study, it was seen that the survival after metastasectomy in rectal cancer is worse than the colon cancer data known from previous studies. This study can be used as data for a more careful selection of the patient group for whom metastasectomy will be performed after conservation therapy and to avoid additional morbidity in patients with borderline values for surgery.
